# Genetic polymorphisms of long noncoding RNA *RP11‐37B2.1* associate with susceptibility of tuberculosis and adverse events of antituberculosis drugs in west China

**DOI:** 10.1002/jcla.22880

**Published:** 2019-03-28

**Authors:** Jiajia Song, Tangyuheng Liu, Zhenzhen Zhao, Xuejiao Hu, Qian Wu, Wu Peng, Xuerong Chen, Binwu Ying

**Affiliations:** ^1^ Department of Laboratory Medicine, West China Hospital Sichuan University Chengdu China; ^2^ Division of Pulmonary Disease, West China Hospital Sichuan University Chengdu China

**Keywords:** adverse drug reaction, lncRNA *RP11‐37B2.1*, single‐nucleotide polymorphisms, susceptibility, tuberculosis

## Abstract

**Background:**

Little knowledge about the biological functions of *RP11‐37B2.1*, a newly defined long noncoding RNA (lncRNA) molecule, is currently available. Previous studies have shown rs160441, located in the *RP11‐37B2.1* gene, is significantly associated with tuberculosis (TB) in a Ghanaian and the Gambian populations.

**Methods:**

We investigated the influence of single‐nucleotide polymorphisms (SNPs) within lncRNA *RP11‐37B2.1* on the risk of TB and the possible correlation with adverse drug reactions (ADRs) from TB treatment in a Western Chinese population. Four SNPs within lncRNA *RP11‐37B2.1* were genotyped in 554 TB cases and 561 healthy subjects using the improved multiplex ligation detection reaction method, and the patients were followed up monthly to monitor the development of ADRs.

**Results:**

No significant association between the SNPs of lncRNA *RP11‐37B2.1* and TB susceptibility was observed (all *P* > 0.05). Surprisingly, significant association was observed between two SNPs (rs218916 and rs160441) and thrombocytopenia development during anti‐TB therapy under the dominant model (*P* = 0.003 and 0.014, respectively).

**Conclusions:**

Our findings firstly exhibit that rs218916 and rs160441 within lncRNA *RP11‐37B2.1* significantly associate with the occurrence of thrombocytopenia and suggest *RP11‐37B2.1* genetic variants are potential biosignatures for thrombocytopenia during anti‐TB treatment.

## 1 INTRODUCTION

Tuberculosis (TB), an ancient infectious disease, infects about a third of the world's population with a yearly incidence of approximately 10 million cases and a mortality of 1.57 million worldwide, based on data from the 2018 World Health Organization (WHO) Global TB report.^1^ Response to the substantial variation between individuals’ susceptibility to the pathogen *Mycobacterium tuberculosis* (MTB),^2^ individuals infected with MTB have a five to ten percent lifetime risk of developing clinical TB.^3^ There is considerable evidence that suggests the host genetics elements play a crucial role in protecting individuals from developing active TB disease.^4^ Twin studies further substantiate the assumption that host genetics greatly contribute to the susceptibility to TB.^5^ Möller et al^6^ demonstrated that the contribution of host hereditary factors to the immune response and phenotypic variation in the population infected with TB ranges up to 71%. However, the exact molecule regulative mechanisms underlying TB remain largely unknown; therefore, further study of the host molecule elements involved in TB infection would be very helpful to understanding the pathogenesis of TB.

Long noncoding RNA (lncRNA) transcripts, the largest species of the nonprotein coding RNAs, have been reported to participate in diverse biological processes and their abnormal expressions have been related to various disease states.^7^ In addition, they are increasingly being recognized to play significant roles in the biological behaviors of TB infection. For example, Yang et al^8^ found aberrant expression of abundant lncRNAs in MTB‐infected macrophages and identified two lncRNAs molecules, *MIR3945HG V1* and *MIR3945HG V2*, which could potentially serve as the promising diagnostic markers for TB. These findings all indicate that lncRNA signatures and their genetic variants hold the potential to behave as the marker for identification of TB infection. *ENSG00000251136* (also known as *RP11‐37B2.1*), located at chromosome8: 89,609,409‐89,757, and 727, adjoining receptor‐interacting serine‐threonine kinase 2 (*RIPK2)* gene, is a newly discovered lncRNA gene. A genome‐wide association study (GWAS) of TB disease conducted by Thye et al^9^ has demonstrated that the single‐nucleotide polymorphism (SNP) rs160441 within the lncRNA *RP11‐37B2.1* gene is significantly associated with genetic predisposition to TB in a Ghanaian population as well as in an independent Gambian population. Due to highly heterogeneity between ethnic groups, specific populations have different causative gene polymorphisms under human pathological conditions, including TB. Inspired predominantly by the previous information, we ultimately selected four SNPs within the lncRNA *RP11‐37B2.1* sequence and investigated their association with the active TB disease risk in a Western Chinese Han population.

In addition to the close relationship between genic polymorphisms and TB susceptibility, series of variations were reported to be related to clinical response to drug therapy in recent years.^10^, ^11^, ^12^ For antituberculosis drugs (ATDs), hepatotoxicity is known as the most serious and prevalent adverse drug reaction. There are a continuously increasing number of identified sequences of polymorphisms related to the occurrence of antituberculosis drug‐induced hepatotoxicity (ATDH): for example, genetic variants within *N‐acetyltransferase 2* (*NAT2*), *nuclear receptor subfamily 1 group I member 2* (*PXR*), and *solute carrier organic anion transporter family member 1B1* (*SLCO1B1*) genes.^13^, ^14^, ^15^ Several published literatures have shown that lncRNAs are significantly associated with the drug effects and resistance in various malignant diseases.^16^, ^17^ For example, in the breast cancer cell experiment, researchers found that downregulation of lncRNA *ROR* inhibited the resistance to Tamoxifen.^17^ Moreover, the GTEx Project shows that the rs160441 and rs218921 within *RP11‐37B2.1* are eQTLs for the *RIPK2*. RIPK2 interaction with NOD2 enhances NF‐kB activity making it an important player in immune response.^18^ Ameliorate acetaminophen (APAP)‐induced liver injury was attenuated by Tovophyllin A by activate Nrf2 and inhibit the NF‐κB signaling pathway.^19^ Therefore, it is speculated that these SNPs within *RP11‐37B2.1* may affect the occurrence of adverse reactions to antituberculosis drugs. In general, TB is a complex disease and immune factors affect its occurrence, development, and even adverse drug reactions. Moreover, no study about the correlation of ATDs with lncRNA molecules has been reported. Thus, the other special purpose of our study was to evaluate possible correlations between lncRNA *RP11‐37B2.1* and ATDs adverse effects.

For all of these reasons, we first evaluated the possible association between four common variations (rs160441， rs218916， rs218921， and rs218936) and TB susceptibility among 554 people with TB and 561 healthy individuals in a Western Chinese Han population in a retrospective study. Then, we explored whether these SNPs had correlations with multiple ATD‐induced adverse reactions (eg, anemia, leukopenia, thrombocytopenia, hepatotoxicity, and kidney damage) in a prospective analysis.

## 2 MATERIALS AND METHODS

### 2.1 Subjects

In our retrospective study, five hundred and fifty‐four cases and five hundred and sixty‐one healthy controls were consecutively recruited between October 2011 and September 2015 from West China Hospital of Sichuan University. All the cases and controls were members of the Chinese Han population. All of the TB patients included were confirmed according to the following criteria: (a) clinically diagnosed by two independent experienced respiratory physicians; (b) positive results of microbiological/pathological examinations (smear/culture/TB‐DNA); (c) positive radiological examination. Patients with evidence of immunodeficiency diseases, diabetes mellitus, and other lung problems were excluded. We recruited the control subjects from healthy blood donors who have no positive TB‐related examinations, no history of TB, and absent symptoms of active TB disease. Clinical data were obtained from qualified interviews or medical records. Participants were interviewed by two experienced visitors with a medical background simultaneously. A 2‐ to 3‐mL peripheral blood sample was collected into EDTA‐anticoagulated tubes from each participant. Genomic DNA was extracted from whole blood samples by the QIAamp^®^ DNA Blood Mini kit (Qiagen, Hilden, Germany) and stored at −80°C for genotyping.

**Figure 1:**
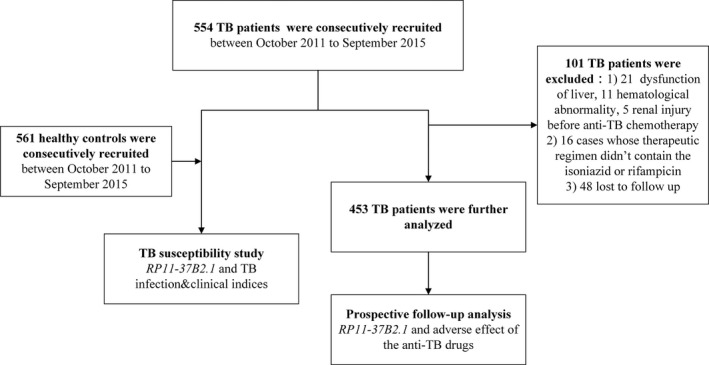
The flow diagram of enrollment of the study participants. There are two parts, TB susceptibility study and prospective follow‐up analysis, in our study. A total of 554 TB patients and 561 healthy control subjects were consecutively recruited in our TB susceptibility study. A total of 453 eligible cases with TB were included in our prospective follow‐up analysis for monitoring the occurrence of adverse drug reaction from ATDs. Annotation: TB, tuberculosis

In our prospective section, TB patients whose ATDs regimens at least included rifampin (RIF, daily 450‐600 mg) and isoniazid (INH, daily 300‐400 mg) for 6 months or more were further selected to monitor the appearance of adverse drug effect from ATDs. This part included the subjects in the previous section without history of liver, kidney, or/and hematologic system disorder before ATDs treatment; additional ineligibility criteria were poor compliance or/and withdrawn during the 6‐month treatment course. Finally, 453 eligible cases with TB were included. We detected peripheral complete blood counts, biochemical examinations, and routine urinalysis monthly for all 453 patients for 6 months or until treatment had been done. ATDs‐induced adverse reactions in this study included anemia, thrombocytopenia, leukopenia, hepatotoxicity, and chronic kidney damage. In terms of hematologic toxicity, hemoglobin‐valley ≤100 g/L, white blood cell count‐valley <3.5 × 10^9^/L, and platelet count‐valley <90 × 10^9^/L were considered to be anemia, leukopenia, and thrombocytopenia, respectively.^20^ Drug‐induced hepatotoxicity was diagnosed according to the criteria of drug‐induced liver disorders in which aspartate aminotransferase (AST) and/or alanine aminotransferase (ALT) levels more than three times the upper limit of normal were considered to have hepatotoxicity.^21^ Chronic kidney injury was diagnosed as persistence of hematuria, proteinuria, or/and casts for more than 90 days.^22^ As you can see in Figure 1, the diagram of study enrollment is shown.

The study was approved by the Committee on Human Research, Publications, and Ethics, West China Hospital, Sichuan University (Reference no. 198; 2014), and all participants or their close relatives obtained the informed consent before blood collection.

### 2.2 Genetic molecular analyses

We obtained genetic variation data of the entire lncRNA*RP11‐37B2.1* locus from the dbSNP database (http://www.ncbi.nlm.nih.gov/projects/SNP/) and comprehensively searched candidate SNPs for this study. We selected SNPs with a minor allele frequency >0.20 according to 1000 Genomes East Asian and their effects on gene expression based on the expression Quantitative Trait Loci (eQTL) information form HaploReg v4.1. Detailed information of candidate SNPs is shown in Tables S1 and S2. In addition, rs160441 was enrolled in this study due to their promising roles in the predisposition to TB based on the genome‐wide association study by Thye et al.^9^ Finally, a total of four SNPs (rs218921, rs160441, rs218916, and rs218936) were selected for subsequent genotyping. Genotyping of these SNPs was performed using an improved multiplex ligation detection reaction (iMLDR) method with the technical support from Shanghai Genesky Biotechnologies Company.^23^ In addition, about ten percent of the total samples were randomly selected for a secondary genotyping, and the coincidence rate of quality control samples was 100%.

### 2.3 Statistical analysis

Statistical analysis was performed with the use of SPSS version 20.0 (IBM, Chicago, USA). The chi‐square test was used for categorical variables, and the Student's *t* test or Mann‐Whitney *U* test for continuous variables was used to analyze the differences in clinical data among the two groups. The goodness‐of‐fit chi‐square test was performed to exclude deviations Hardy‐Weinberg equilibrium (HWE) for controls. The odds ratios (ORs) and 95% confidence intervals (CIs) were calculated by unconditional logistic regression analysis using PLINK version 1.07^24^; the linkage disequilibrium (LD) was estimated by calculating the pairwise *r* ^2^ coefficient. Haplotype analysis was performed by Haploview software version 4.2, which employed the expectation‐maximization clustering algorithm. Prior to data collection, PASS Statistical Software v11 was used to perform power calculations. All tests were two‐sided, and *P* < 0.05 was considered to be statistical significance; PhenoSpD tool was adopted to correct for multiple testing.^25^ R code and documentation for PhenoSpD V1.0.0 is available at https://github.com/MRCIEU/PhenoSpD.

## 3 RESULTS

### 3.1 General characteristics of the Western Chinese Han population

We studied 1115 Western Chinese Han individuals including 554 TB patients and 561 controls (Basic data were shown in Table S3). No statistically significant differences were observed for age and gender between two groups. Significant differences in smoking status, Bacillus Calmette‐Guerin (BCG) scar, and body mass index (BMI) were observed between the two groups, with smoking and having a BCG scar being more prevalent in the case group (both *P* < 0.001). From Table S3, TB patients had significantly higher levels of C‐reactive protein (CRP), erythrocyte sedimentation rate (ESR), leukocytes (WBC), platelets (PLT), and monocytes, whereas the TB patients presented an obvious reduction in the indices of albumin, erythrocytes, and hemoglobin (Hb) compared with the healthy group (*P* < 0.001 for all).

In our prospective study, all included 453 patients underwent laboratory routine testing before the anti‐TB treatment and then consecutively received these tests monthly. Quantitative results of laboratory examinations of TB patients at peak or valley during anti‐TB therapy are as follows: Hb‐valley (mean [interquartile range]): 118 (100‐134) g/L, PLT‐valley: 213 (149‐288) × 10^9^/L, WBC‐valley: 4.97 (3.89‐6.33) × 10^9^/L, total bilirubin (TBIL)‐peak: 9.5 (6.4‐15.6) μmol/L, ALT‐peak: 32 (16‐66) IU, creatinine‐peak: 62.3 (51.0‐74.0) μmol/L, and URIC‐peak 283 (202‐382) μmol/L. In our analyses, leukopenia (16.11%, 73/453) was the most common adverse event, followed by hepatotoxicity (12.36%, 56/453) and anemia (8.39%, 38/453). No patient in our case cohort had chronic kidney damage.

### 3.2 Association of lncRNA *RP11‐37B2.1* genetic polymorphisms with susceptibility to TB

The genotype distributions of the tested SNPs in the control group were consistent with the HWE (*P* > 0.05). TB patients and the controls had very similar genotype and allele distributions among all four SNPs (*P* > 0.05) in Table S4. Frustratingly, these four SNPs had nothing to do with predisposition to TB under three genetic patterns (*P* > 0.05) in Table S5. Furthermore, we also conducted the age‐subgroup and clinical subtype‐subgroup analysis according to the earlier studies^26^, ^27^ and determined these four candidate SNPs were not correlated with specific age group and specific tubercular subtype (data not shown).

All four variants in the lncRNA *RP11‐37B2.1* gene were analyzed (results showed in Figure S1) whether were in the linkage disequilibrium block according to the threshold of pairwise *r* ^2^ > 0.5. Six haplotypes, CCC, TTT, CTT, CTC, CCT, and TTC, were constructed for lncRNA *RP11‐37B2.1*, which consisted of rs160441, rs218916, and rs218936. Table S6 concluded the haplotype frequencies and their associations with tuberculosis susceptibility. The results revealed that no haplotype was significantly associated with TB tuberculosis predisposition.

### 3.3 Association of lncRNA *RP11‐37B2.1* genetic polymorphisms with ATD‐induced adverse reactions

In this prospective part, we compared the incidences of ATD‐induced adverse reactions in different genotypes. Although we failed to observe any significant associations between these four lncRNA *RP11‐37B2.1* genetic polymorphisms and TB risk, TB non‐susceptibility loci posed the associations with the occurrence of drug‐induced thrombocytopenia. Rs218916 is shown to be closely correlated with the presence of drug‐induced thrombocytopenia by applying the dominant model (*P* = 0.003). The results suggested that the T alleles of rs218916 might serve as a hazard for thrombocytopenia induced by ATDs (OR = 5.32, 95% CI = 1.54‐18.32 in Table 1). As for rs160441 and rs218936, patients carrying T allele‐involving genotypes would have more chance to have thrombocytopenia arising from anti‐TB chemotherapy treatment than CC genotype carriers with the estimated *P = *0.014 (OR = 3.18, 95% CI = 1.21‐8.37, presented in Table 2) and *P* = 0.018 (OR = 3.23, 95% CI = 1.16‐8.97, presented in Table 3), respectively. Also, weak correlation was found between rs218921 and anti‐TB drug‐induced hepatotoxicity in the dominant model (*P* = 0.048, in Table 4).

**Table 1:** Association of rs218916 polymorphism with adverse drug reactions from TB patients in dominant model

Drug adverse reactions	CC (n = 206)	CT + TT (n = 247)	*P*	OR (95% CI)
Anemia n (%)	14 (6.80)	24 (9.72)	0.264	1.48 (0.73‐2.93)
Leukopenia n (%)	34 (16.50)	39 (15.79)	0.837	0.95 (0.57‐1.57)
Thrombocytopenia n (%)	3 (1.46)	18 (7.29)	0.003	5.32 (1.54‐18.32)
Hepatotoxicity n (%)	23 (11.17)	33 (13.36)	0.480	1.23 (0.70‐2.17)

CI, confidence interval; OR, odds ratio; SNP, single‐nucleotide polymorphism.

*P* values were adjusted by age and gender.

**Table 2:** Association of rs160441 polymorphism with adverse drug reactions from TB patients in dominant model

Drug adverse reactions	CC (n = 248)	CT + TT (n = 205)	*P*	OR (95% CI)
Anemia n (%)	20 (8.06)	18 (8.78)	0.784	1.10 (0.57‐2.14)
Leukopenia n (%)	40 (16.13)	33 (16.10)	0.993	1.00 (0.60‐1.65)
Thrombocytopenia n (%)	6 (2.42)	15 (7.32)	0.014	3.18 (1.21‐8.37)
Hepatotoxicity n (%)	31 (12.50)	25 (12.20)	0.922	0.97 (0.55‐1.71)

CI, confidence interval; OR, odds ratio; SNP, single‐nucleotide polymorphism.

*P* values were adjusted by age and gender.

**Table 3:** Association of rs218936 polymorphism with adverse drug reactions from TB patients in dominant model

Drug adverse reactions	CC (n = 222)	CT + TT (n = 231)	*P*	OR (95% CI)
Anemia n (%)	20 (9.01)	18 (7.79)	0.640	0.84 (0.44‐1.66)
Leukopenia n (%)	38 (17.12)	35 (15.15)	0.569	0.87 (0.52‐1.43)
Thrombocytopenia n (%)	5 (2.25)	16 (6.92)	0.018	3.23 (1.16‐8.97)
Hepatotoxicity n (%)	26 (11.71)	30 (12.99)	0.680	1.23 (0.64‐1.97)

CI: confidence interval; OR: odds ratio; SNP: single‐nucleotide polymorphism.

*P* values were adjusted by age and gender.

**Table 4:** Association of rs218921 polymorphism with adverse drug reactions from TB patients in dominant model

Drug adverse reactions	CC + CT(n = 242)	TT (n = 211)	*P*	OR (95% CI)
Anemia n (%)	21 (8.68)	17 (8.06)	0.812	0.922 (0.47‐1.80)
Leukopenia n (%)	41 (16.94)	32 (15.17)	0.608	0.88 (0.53‐1.45)
Thrombocytopenia n (%)	14 (5.79)	7 (3.32)	0.213	0.56 (0.22‐1.41)
Hepatotoxicity (%)	23 (9.50)	33 (15.64)	0.048	1.77 (1.00‐3.12)

CI: confidence interval; OR: odds ratio; SNP: single‐nucleotide polymorphism.

*P* values were adjusted by age and gender.

We have adopted PhenoSpD tool to estimate phenotypic correlation and correct for multiple testing.^25^ Our effective number of independent variables is 3, and the experiment‐wide significance threshold required to keep type I error rate at 5% is 0.0170 according to PhenoSpD correction. After PhenoSpD correction, the correlation between the SNPs (rs218916 and rs160441) and the occurrence of drug‐induced thrombocytopenia was still statistically significant, while rs218936 was not. The correlation between rs218921 and anti‐TB drug‐induced hepatotoxicity risk was not statistically significant too after PhenoSpD correction.

Moreover, the association was observed between the haplotypes TTC consisted of rs160441, rs218916, and rs218936 and ATD‐induced thrombocytopenia (*P* = 0.019, OR = 4.31, 95% CI = 1.15‐16.19). The association was not observed between other haplotypes (CCC, TTT, CTT, CTC, and CCT) consisted of these SNPs and ATD‐induced thrombocytopenia (data not shown).

## 4 DISCUSSION

Over the last two decades, accumulating evidence indicates that lncRNAs might modulate the innate immune response.^28^, ^29^ More and more lncRNAs were determined, such as *lnc‐interleukin 7 receptor*, *nonprotein coding RNA repressor of NFAT (NRON)*, and many more, representing a new series of molecules that is associated with the gene expressions and functions of immune cells.^28^, ^29^, ^30^ Recently, lncRNAs have key functions in ward off MTB invasion. Aberrant expressions in cells infected with MTB provide promising biomarkers for diagnosis and/or prognosis. For instance, a research by Fu et al uncovered that *suppressor of cytokine signaling 3 (SOCS3)*, which is an essential negative regulator of cytokine defense to Mycobacterium tuberculosis invasion and its nearby lncRNA *XLOC_012582*, was overexpressed in B cells from active TB patients.^31^ On the other hand, there are limited data support the association between lncRNA genetic polymorphisms and susceptibility and phenotypes of TB. The TB GWAS conducted by Thye^9^ showed that one SNP in the lncRNA *RP11‐37B2.1*, rs160441, was significantly associated with TB in a Ghanaian population and a Gambian population, but Qinying Hu^32^ and our study found no association between the polymorphism rs160441 and TB susceptibility in the Chinese population. The heterogeneity may be due to that study populations were from different ancestry. Similar to our results, the recent GWAS of Curtis et al discovered a series of new susceptibility loci at 8q24 to TB in a Russians population and reported that the SNP rs10956514 was a functional locus,^33^ but Miao et al found rs10956514 did not contribute to TB susceptibility in a Chinese population.^34^ There are also several other likely sources of heterogeneity besides population, including phenotype definition, ascertainment, strain of M. tuberculosis infecting the population, and differences in genotyping and statistical methods.^35^

Except for the incidence of TB, ATD adverse reactions are an important part which contribute toward anti‐TB treatment discontinuation or failure. Although drug‐induced liver injury is the most general and well‐studied adverse reaction caused by TB therapy with INH and RFP,^36^ thrombocytopenia is less common but far more likely to be fatal adverse effect seen with certain ATDs. RFP is the agent most commonly associated with ATD‐induced thrombocytopenia.^37^ INH‐induced thrombocytopenia is a rare presentation, and only a few such cases have been reported in the literature.^38^ We first identified that three SNPs (rs160441, rs218936, and rs218916) within lncRNA *RP11‐37B2.1* might be associated with drug‐induced thrombocytopenia before PhenoSpD correction. The association between two SNPs (rs160441, rs218936) and thrombocytopenia were weak; therefore, we speculated this association may be influenced by the result of LD between three SNPs. In our work, we observed the potential association between the haplotypes TTC consisted of rs160441, rs218916, and rs218936 and ATD‐induced thrombocytopenia (*P* = 0.019, OR = 4.306, 95% CI = 1.15‐16.19). We consider haplotype TTC as promising marker for predict the risk of thrombocytopenia. LncRNA *RP11‐37B2.1* adjoins *RIPK2*, which is a component of signaling complexes in both the innate and adaptive immune pathways. A study has shown that RFP causes thrombocytopenia by Immunization. After combining with some molecules in the plasma, RFP can act as a hapten and stimulate antibodies. When RFP appears again in the plasma, these antibodies are believed to fix a complement on the platelets, resulting in platelet destruction.^39^We can speculate that these loci in lncRNA *RP11‐37B2.1* may affect the incidence rate of thrombocytopenia by immunity. These results indicate that the variants of lncRNA *RP11‐37B2.1* contribute to host response to drug treatment; however, the mechanism of how they affect drug adverse reactions remains unclear. Thus, more rigorous research at the molecular gene level should be conducted.

There are several limitations to this study. First, our sample size is limited, which leads to a higher false‐negative rate. Second, the only gene determinants are not sufficient to trigger ATD adverse reactions, and combination of nongenetic and genetic risk factors may be more potent in predicting ATD adverse reactions. Third, according to eQTL analysis, the rs160441 and rs218921 are eQTLs for both *RP11‐37B2.1* and *RIPK2*. Therefore, in the target tissue of TB infection *RIPK2* is as good candidate as *RP11‐37B2.1* in the future studies. Therefore, better replications in other large independent populations and ethnicities are urgently needed to conclusively confirm or reject our findings.

## 5 CONCLUSIONS

No significant association between the SNPs of lncRNA *RP11‐37B2.1* and TB susceptibility was observed in our study. However, our findings firstly exhibit that rs218916 and rs160441 within lncRNA *RP11‐37B2.1* significantly associate with the occurrence of thrombocytopenia and suggest *RP11‐37B2.1* genetic variants are potential biosignatures for thrombocytopenia during anti‐TB treatment.
