# Supplementary material for: Genetic polymorphisms of long noncoding RNA RP11‐37B2.1 associate with susceptibility of tuberculosis and adverse events of antituberculosis drugs in west China
Source: J Clin Lab Anal. 2019 Mar 28;33(5):e22880. doi: 10.1002/jcla.22880 (PMC6595342; doi:10.1002/jcla.22880)
Supplement: Supplementary file 1 [file JCLA-33-e22880-s001.docx]

Supplementary Table 1. Characteristics of the studied 4 SNPs.

| **SNP** | **Chr: position** | **MAF** | **eQTL** |  |
| --- | --- | --- | --- | --- |
|  |  |  | **Correlated gene** | |
| rs160441 | 8: 90656988 | 0.25 | *RP11-37B2.1，RIPK2* | |
| rs218916 | 8: 90700937 | 0.33 | *RP11-37B2.1，ENSG00000251136* | |
| rs218921 | 8: 90632741 | 0.32 | *RIPK2* | |
| rs218936 | 8: 90755414 | 0.305 | *RP11-37B2.1，ENSG00000251136* | |

SNP: single-nucleotide polymorphism; Chr: chromosome; MAF: minor allele frequency; eQTL: expression Quantitative Trait Loci.

Supplementary Table 2. Analyses of Expression quantitative trait locus (eQTL) in the studied 4 SNPs.

| SNP | Tissue | Correlated gene | *P* |
| --- | --- | --- | --- |
| rs160441 | Skin_Sun_Exposed_Lower_leg | *RP11-37B2.1* | 2.97×10^-24^ |
|  | Adipose_Subcutaneous | *RP11-37B2.1* | 1.01×10-^14^ |
|  | Nerve_Tibial | *RP11-37B2.1* | 1.63×10^-14^ |
|  | Lung | *RP11-37B2.1* | 7.93×10^-14^ |
|  | Artery_Tibial | *RP11-37B2.1* | 1.15×10^-10^ |
|  | Breast_Mammary_Tissue | *RP11-37B2.1* | 4.13×10^-10^ |
|  | Adipose_Visceral_Omentum | *RP11-37B2.1* | 2.13×10^-8^ |
|  | Cells_Transformed_fibroblasts | *RP11-37B2.1* | 9.40×10^-8^ |
|  | Esophagus_Muscularis | *RP11-37B2.1* | 9.86×10^-8^ |
|  | Artery_Coronary | *RP11-37B2.1* | 1.71×10^-7^ |
|  | Thyroid | *RP11-37B2.1* | 6.02×10^-7^ |
|  | Pancreas | *RP11-37B2.1* | 4.81×10^-6^ |
|  | Esophagus_Mucosa | *RP11-37B2.1* | 7.34×10^-6^ |
|  | Whole_Blood | *RIPK2* | 5.81×10^-26^ |
| rs218916 | Skin_Sun_Exposed_Lower_leg | *RP11-37B2.1* | 1.47×10^-23^ |
|  | Adipose_Subcutaneous | *RP11-37B2.1* | 1.06×10^-22^ |
|  | Nerve_Tibial | *RP11-37B2.1* | 1.45×10^-16^ |
|  | Breast_Mammary_Tissue | *RP11-37B2.1* | 2.28×10^-13^ |
|  | Lung | *RP11-37B2.1* | 9.93×10^-13^ |
|  | Cells_Transformed_fibroblasts | *RP11-37B2.1* | 3.23×10^-12^ |
|  | Artery_Tibial | *RP11-37B2.1* | 5.01×10^-12^ |
|  | Thyroid | *RP11-37B2.1* | 1.29×10^-11^ |
|  | Adipose_Visceral_Omentum | *RP11-37B2.1* | 2.03×10^-11^ |
|  | Esophagus_Muscularis | *RP11-37B2.1* | 1.11×10^-7^ |
|  | Pancreas | *RP11-37B2.1* | 1.16×10^-7^ |
|  | Artery_Coronary | *RP11-37B2.1* | 1.59×10^-7^ |
|  | Small_Intestine_Terminal_Ileum | *RP11-37B2.1* | 1.21×10^-6^ |
|  | Artery_Aorta | *RP11-37B2.1* | 1.24×10^-6^ |
|  | Stomach | *RP11-37B2.1* | 1.51×10^-6^ |
|  | Colon_Transverse | *RP11-37B2.1* | 2.40×10^-6^ |
|  | Spleen | *RP11-37B2.1* | 1.02×10^-5^ |
|  | Esophagus_Mucosa | *RP11-37B2.1* | 1.32×10^-5^ |
|  | Lymphoblastoid_EUR_genelevel | *ENSG00000251136* | 3.90×10^-6^ |
| rs218921 | Whole_Blood | *RIPK2* | 9.19×10^-5^ |
| rs218936 | Skin_Sun_Exposed_Lower_leg | *RP11-37B2.1* | 4.97×10^-26^ |
|  | Adipose_Subcutaneous | *RP11-37B2.1* | 1.02×10^-22^ |
|  | Nerve_Tibial | *RP11-37B2.1* | 3.79×10^-18^ |
|  | Artery_Tibial | *RP11-37B2.1* | 2.93×10^-15^ |
|  | Thyroid | *RP11-37B2.1* | 3.21×10^-14^ |
|  | Lung | *RP11-37B2.1* | 6.65×10^-14^ |
|  | Cells_Transformed_fibroblasts | *RP11-37B2.1* | 1.19×10^-13^ |
|  | Breast_Mammary_Tissue | *RP11-37B2.1* | 1.42×10^-13^ |
|  | Adipose_Visceral_Omentum | *RP11-37B2.1* | 3.31×10^-13^ |
|  | Stomach | *RP11-37B2.1* | 5.46×10^-8^ |
|  | Colon_Transverse | *RP11-37B2.1* | 6.17×10^-8^ |
|  | Esophagus_Muscularis | *RP11-37B2.1* | 6.93×10^-8^ |
|  | Artery_Coronary | *RP11-37B2.1* | 2.62×10^-7^ |
|  | Pancreas | *RP11-37B2.1* | 4.63×10^-7^ |
|  | Whole_Blood | *RP11-37B2.1* | 6.45×10^-7^ |
|  | Skin_Not_Sun_Exposed_Suprapubic | *RP11-37B2.1* | 6.83×10^-7^ |
|  | Esophagus_Mucosa | *RP11-37B2.1* | 8.53×10^-7^ |
|  | Artery_Aorta | *RP11-37B2.1* | 1.10×10^-6^ |
|  | Adrenal_Gland | *RP11-37B2.1* | 2.38×10^-6^ |
|  | Esophagus_Gastroesophageal_Junction | *RP11-37B2.1* | 3.12×10^-6^ |
|  | Lymphoblastoid_EUR_genelevel | *ENSG00000251136* | 1.33×10^-7^ |

Data source: Genotype-Tissue Expression (GTEx) Project

| Supplementary Table 3. Demographic and clinical characteristics of study participants in Western China. | | | |
| --- | --- | --- | --- |
| **Characteristics** | **TB (n = 554)** | **HC (n = 561)** | ***P*** |
| **General data** |  |  |  |
| Age, mean ± SD (years) | 43.25 ± 19.87 | 44.23 ± 11.49 | 0.417 |
| Male/female | 343/211 | 331/230 | 0.351 |
| BMI (kg/m^2^) | 20.74 ± 2.53 | 23.53 ± 2.31 | **<0.001** |
| BCG scar n (%) |  |  | **<0.001** |
| Yes | 289 (52.17) | 240 (42.78) |  |
| No | 200 (36.10) | 250 (44.56) |  |
| Uncertain | 65 (11.73) | 71 (12.66) |  |
| Smoking n (%) |  |  | **<0.001** |
| Smoking | 299 (53.97) | 224 (39.93) |  |
| Ever Smoking | 94 (16.97) | 95 (16.93) |  |
| Nonsmoking | 161 (29.06) | 242 (43.14) |  |
| **TB subtype n (%)** |  |  |  |
| PTB | 275 (49.64) | **-** | **-** |
| EPTB | 62 (11.19) | **-** | **-** |
| PTB & EPT | 217 (39.17) | **-** | **-** |
| **Laboratory examinations** | | | |
| Albumin (g/L) | 34.55 ± 6.65 | 46.72 ± 2.60 | **<0.001** |
| Erythrocyte (×10^12^/L) | 4.14 ± 0.79 | 4.85 ± 0.46 | **<0.001** |
| Hemoglobin (g/L) | 115.85 ± 23.47 | 147.26 ± 15.13 | **<0.001** |
| Platelets (×10^9^/L) | 250.40 ± 68.10 | 171.12 ± 49.07 | **<0.001** |
| Leucocytes (×10^9^/L) | 7.57 ± 3.49 | 6.11 ± 1.30 | **<0.001** |
| Monocytes (×10^9^/L) | 0.62 ± 0.35 | 0.35 ± 0.12 | **<0.001** |
| C-reactive protein (mg/L) | 19.65 (6.04-69.13) | 5.43 (1.79-18.42) | **<0.001** |
| ESR (mm/h) | 46.00 (22.00-74.50) | 20.36 (8.45-56.81) | **<0.001** |
| Positive TB-DNA n (%) | 212 (38.26) | **-** | **-** |
| Positive smear n (%) | 162 (29.24) | **-** | **-** |
| Positive culture n (%) | 44 (7.94) | **-** | **-** |
| **Main alteration of CT n(%)** |  |  |  |
| Normal | 8 (1.44) | **-** | **-** |
| Infiltration and effusion | 233 (42.06) | **-** | **-** |
| Proliferation and consolidation | 79 (14.26) | **-** | **-** |
| Caseation and cavitation | 112 (20.22) | **-** | **-** |
| Fibrosis and calcification | 122 (22.02) | - | - |

TB: tuberculosis; HC: healthy controls; SD: standard deviation; BMI: body mass index; BCG: Bacillus Calmette-Guerin; PTB: pulmonary tuberculosis; EPTB: extra-pulmonary tuberculosis; PTB & EPTB: pulmonary tuberculosis combined with extra-pulmonary tuberculosis; ESR: erythrocyte sedimentation rate; CT: computed tomography. *P* significant associations were denoted in bold

Supplementary Table 4. Genotype distributions of lncRNA *RP11-37B2.1* polymorphisms of TB cases and healthy controls**.**

| **SNP** |  | **Case n(%)** | **Control n(%)** | **OR (95% CI)** | ***P*** |  | **Case n(%)** | **Control n(%)** | ***P*** |
| --- | --- | --- | --- | --- | --- | --- | --- | --- | --- |
| rs218916 | T | 356(32.13) | 362(32.26) | 0.99(0.83-1.19) | 0.994 | TT | 57 (10.29) | 56 (9.98) | 0.954 |
| C>T | C | 752(67.87) | 760(67.74) |  |  | TC | 242 (43.68) | 250 (44.56) |  |
|  |  |  |  |  |  | CC | 255 (46.03) | 255 (45.45) |  |
| rs160441 | T | 279(25.18) | 276(24.6) | 1.03(0.85-1.25) | 1.032 | TT | 33 (5.96) | 34 (6.06) | 0.894 |
| C>T | C | 829(74.82) | 846(75.4) |  |  | TC | 213 (38.45) | 208 (37.08) |  |
|  |  |  |  |  |  | CC | 308 (55.60) | 319 (56.86) |  |
| rs218921 | C | \| 348(31.41) \| \| --- \| | 372(33.16) | 0.92(0.77-1.10) | 0.923 | CC | 50 (9.03) | 68 (12.12) | 0.223 |
| T>C | T | 760(68.59) | 750(66.84) |  |  | CT | 248 (44.77) | 236 (42.07) |  |
|  |  |  |  |  |  | TT | 256 (46.21) | 257 (45.81 ) |  |
| rs218936 | T | 329(29.69) | 351(31.28) | 0.93(0.78-1.11) | 0.928 | TT | 48 (8.66) | 58 (10.34) | 0.621 |
| C>T | C | 779(70.31) | 771(68.72) |  |  | TC | 233 (42.06) | 235 (41.89) |  |
|  |  |  |  |  |  | CC | 273 (49.28) | 268 (47.78) |  |

SNP: single-nucleotide polymorphism; OR: odds ratio; CI: confidence interval.

Supplementary Table 5. Comparison of lncRNA *RP11-37B2.1* polymorphisms in relation to TB risk in Chinese Han population in the genetic models (additive, dominant, and recessive model).

| **SNP** | **Additive Model** | |  | **Dominant Model** | |  | **Recessive Model** | |
| --- | --- | --- | --- | --- | --- | --- | --- | --- |
|  | **OR (95% CI)** | ***P*** |  | **OR (95% CI)** | ***P*** |  | **OR (95% CI)** | ***P*** |
| rs218921T>C | 0.99(0.83-1.19) | 0.946 |  | 0.98(0.77-1.24) | 0.847 |  | 1.0(0.70-1.53) | 0.865 |
| rs160441C>T | 1.03(0.85-1.25) | 0.750 |  | 1.05(0.83-1.33) | 0.670 |  | 0.98(0.60-1.61) | 0.942 |
| rs218916C>T | 0.92(0.77-1.10) | 0.380 |  | 0.98(0.78-1.24) | 0.894 |  | 0.72(0.49-1.06) | 0.094 |
| rs218936C>T | 0.93(0.78-1.11) | 0.417 |  | 0.94(0.74-1.19) | 0.614 |  | 0.82(0.55-1.23) | 0.341 |

Dominant model: variant-containing genotypes vs. homozygous wild-type genotype; Recessive model: homozygous variant genotype vs. wild-type-containing genotype; SNP: single-nucleotide polymorphism; OR: odds ratio; CI: confidence interval.

Supplementary Table 6. Haplotype constructions of the lncRNA *RP11-37B2.1* variants associated with the risk of tuberculosis. Six haplotypes, CCC, TTT, CTT, CTC, CCT, and TTC, were constructed for lncRNA RP11-37B2.1, which consisted of rs160441, rs218916, and rs218936.

| **Haplotype** | **Haplotype Frequency** | | ***P*** | **OR(95%CI)** |
| --- | --- | --- | --- | --- |
|  | **Case** | **Control** |  |  |
| CCC | 0.655 | 0.649 | - | 1.00 |
| TTT | 0.229 | 0.231 | 0.87 | 0.98 (0.80 - 1.21) |
| CTT | 0.050 | 0.058 | 0.40 | 0.85 (0.58 - 1.24) |
| CTC | 0.026 | 0.024 | 0.76 | 1.09 (0.63 - 1.89) |
| CCT | 0.018 | 0.024 | 0.37 | 0.77 (0.43 - 1.37) |
| TTC | 0.018 | 0.010 | 0.19 | 1.66 (0.78 - 3.51) |


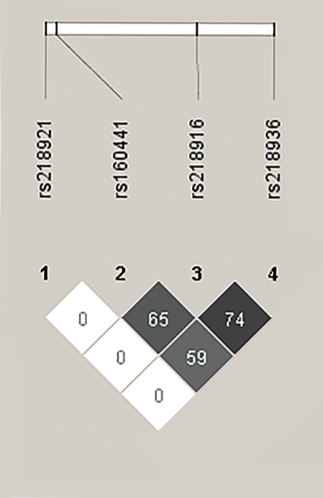


Supplementary Figure 1. Linkage disequilibrium (LD) plot of four SNPs of lncRNA *RP11-37B2.1*. Strong LD is represented by a high linkage disequilibrium correlation coefficient and a darker square.
